# Synaptic mechanisms for differential severity of social preference deficits in male and female mice induced by diminished activity-dependent BDNF

**DOI:** 10.3389/fnins.2026.1864866

**Published:** 2026-07-27

**Authors:** Kaijie Ma, Maria Webb, Samuel S. Newton, Francis S. Lee, Luye Qin

**Affiliations:** 1 Division of Biomedical and Translational Sciences, Sanford School of Medicine, University of South Dakota, Vermillion, SD, United States; 2 School of Health Sciences, University of South Dakota, Vermillion, SD, United States; 3 Department of Psychiatry, Sackler Institute for Developmental Psychobiology, Weill Cornell Medicine, New York, NY, United States; 4 Department of Pharmacology, Sackler Institute for Developmental Psychobiology, Weill Cornell Medicine, New York, NY, United States

**Keywords:** activity-dependent BDNF signaling, neuronal excitability, sex, synaptic transmission, Val66Met polymorphism

## Abstract

Males are more commonly diagnosed with autism spectrum disorder (ASD) than females with a ratio of about 4–1. However, the neural mechanisms underlying the sex differences in ASD are unknown. Social deficits are the core symptoms of patients with ASD. Previous studies showed that diminished activity-dependent brain-derived neurotrophic factor (BDNF) signaling induced differential severity of autism-like social preference deficits in male and female mice by using a mouse model with genetic knock-in of human BDNF methionine (Met) allele, which significantly decreased activity-dependent BDNF release without affecting basal BDNF secretion. Here, we investigated the synaptic mechanisms for diminished activity-dependent BDNF-induced differential severity of social preference deficits in males and females. The prefrontal cortex (PFC) is a critical brain region for social behaviors. Whole-cell patch-clamp brain slice recordings showed that diminished activity-dependent BDNF signaling differentially increased the frequency of spontaneous action potentials (sAPs) of pyramidal neurons in the PFC of male and female BDNF^+/Met^ mice. The frequency of sAPs in male BDNF^+/Met^ mice was higher than in female BDNF^+/Met^ mice. Diminished activity-dependent BDNF signaling differentially enhanced excitatory synaptic transmission and dampened inhibitory synaptic transmission of pyramidal neurons at pre- and post- synapses in males and females, which were mediated by dysregulated transcriptional levels of key synaptic genes. Chemogenetic inhibition of pyramidal neurons in the PFC of BDNF^+/Met^ mice was sufficient to ameliorate autism-like social preference deficits in males and females. This study reveals synaptic mechanisms underlying the differential severity of social preference deficit in male and female BDNF^+/Met^ mice, which provides a potential neural basis for sex differences in male and female ASD patients with and without the BDNF Val66Met SNP.

## 1 Introduction

According to the newest estimates from the CDC (Centers for Disease Control and Prevention)’s ADDM (Autism and Developmental Disabilities Monitoring) Network (2025), 1 in 31 (3.2%) children aged 8 years have been diagnosed with autism spectrum disorder (ASD) (surveillance year 2022). Human neuroimaging studies from ASD patients showed differences in brain morphology, function, and circuits connectivity in males and females (Mo et al., 2021), which may underlie the heterogeneity of phenotypic manifestations in males and females. However, the potential neural mechanisms underlying the sex differences in ASD remain a significant gap (Shansky and Woolley, 2016).

ASD is caused by genetic mutations and/or exposure to environmental factors (Simons Foundation Autism Research Initiative, SFARI) (Satterstrom et al., 2020; Tammimies et al., 2015; Wang T. et al., 2016; De Rubeis et al., 2014; Iossifov et al., 2015; Faundes et al., 2018; Stessman et al., 2017; Karimi et al., 2017). We and others believe that diminished activity-dependent neural signaling is a common molecular pathway dysregulated in patients with ASD (Ebert and Greenberg, 2013; Yap and Greenberg, 2018). Given the critical role of activity-dependent brain-derived neurotrophic factor (BDNF) signaling in the brain, the impact of diminished activity-dependent BDNF signaling on social deficits in males and females by using mice with genetic knock-in of this human BDNF methionine (Met) allele was examined, which significantly reduced up to 30% of activity-dependent BDNF release without affecting basal BDNF secretion (Chen et al., 2006; Egan et al., 2003). Diminished activity-dependent BDNF signaling caused differential severity of autism-like social preference deficits in male and female mice, and male mice appeared to be more severe than females (Ma et al., 2023).

Growing evidence suggests that elevated excitatory/inhibitory (E/I) balance in the PFC is a major pathogenesis of social deficits in animal autistic mouse models and ASD patients (Lee et al., 2016; Gao and Penzes, 2015; Yizhar et al., 2011). Pyramidal neurons are the principal neurons in the prefrontal cortex (PFC), a critical brain region for social behavior (Arnsten and Rubia, 2012; Davidson, 2002; Qin et al., 2019b), which are impaired in patients with ASD (Stoner et al., 2014). Activity-dependent BDNF signaling plays critical roles in cognition via the homeostatic regulation of neuronal intrinsic properties and synaptic transmission (Song et al., 2017; Greenberg et al., 2009; Chao, 2003). The diminished activity-dependent BDNF signaling increased intrinsic excitability of pyramidal neurons in male BDNF^+/Met^ mice, but not in female BDNF^+/Met^ mice, via increased expression of *Scn2a* (encodes sodium channel Nav1.2) and decreased expression of *Kcnn2* (encodes small-conductance calcium-activated potassium channel 2, SK2) (Ma et al., 2024a), which decreased the thresholds of action potentials and facilitated the propagation of firing (Sanders et al., 2018; Spratt et al., 2019; Faber and Sah, 2007). The increased intrinsic excitability of pyramidal neurons in the PFC might partially contribute to social preference deficits in male BDNF^+/Met^ mice. However, the neural mechanisms that contribute to social preference deficits in female BDNF^+/Met^ mice are unknown. The driving force for neuronal excitability is derived from intrinsic membrane properties (permissive gate) and synaptic inputs (Pratt and Aizenman, 2007). In this study, we examined the impact of activity-dependent BDNF signaling on the synaptic transmission of pyramidal neurons in the PFC to reveal the potential neural mechanisms underlying the differential severity of social preference deficit in male and female BDNF^+/Met^ mice.

## 2 Materials and methods

### 2.1 Animal care and husbandry

The use of animals and procedures performed were approved by the Institutional Animal Care and Use Committee of Sanford School of Medicine, University of South Dakota. A mouse model with genetic knock-in of a human BDNF Met variant was created, and the procedures for heterozygote breeding and genotyping were described previously (Chen et al., 2006). These mice were backcrossed more than 12 generations into the C57BL/6 strain. Animals were group-housed (*n* = 4–5/cage) in standard cages and were kept on a 12-h light–dark cycle in a temperature-controlled room. Food and water were available *ad libitum*. Experiments were performed in male and female BDNF^+/Met^ mice and sex- and age- matched WT littermates BDNF*^+/+^*, which were derived from heterozygous BDNF^+/Met^ breeding pairs. All assays were performed when mice were 6–8 weeks old. Experiments were carried out by investigators in a blind fashion (with no prior knowledge of genotypes).

### 2.2 Brain slice preparation

Coronal brain slices containing PFC were prepared from 2-month-old male and female BDNF*^+/+^* and BDNF^+/Met^ mice as described previously (Qin et al., 2019b; Ma et al., 2024a; Qin et al., 2018).

### 2.3 Whole-cell patch-clamp brain slice recordings

For recordings, the brain slice was positioned in a perfusion chamber attached to the fixed stage of an upright microscope (Olympus) and submerged in continuously flowing oxygenated ACSF (in mM: 130 NaCl, 26 NaHCO_3_, 1 CaCl_2_, 5 MgCl_2_, 3 KCl, 1.25 NaH_2_PO4, 10 glucose, pH 7.4, 300 mOsm). Layer V pyramidal neurons in the PFC were visualized with infrared differential interference contrast video microscopy. Recordings were performed with a multi-Clamp 700B amplifier (Molecular Devices), and data were acquired using pClamp 11.2 software, filtered at 1 kHz, and a sampling rate at 10 kHz with an Axon Digidata 1550B plus HumSilencer digitizer (Molecular Devices). Recording electrodes were pulled from borosilicate glass capillaries (1.5/0.86 mm OD/ID) with a micropipette puller (Sutter Instrument, model P-97, Novato, CA). The resistances of patch electrodes were 4–6 MΩ when filled with internal solution.

To measure spontaneous action potentials, the brain slice was bathed in a modified ACSF with low (0.5 mM) MgCl_2_ to elevate neuronal activity, which more closely mimicked the ionic composition of the brain interstitial fluid *in situ* (Qin et al., 2021). The pipettes were filled with an intracellular solution (in mM): 124 K-gluconate, 1 MgCl_2_, 6 KCl, 5 EGTA, 10 HEPES, 0.5 CaCl_2_, and 12 phosphocreatine, 5 MgATP, 0.5 Na_2_GTP, 0.2 Leupeptin, pH 7.2–7.3, 265–270 mOsm.

For spontaneous excitatory postsynaptic current (sEPSC) recording, neurons were held at −70 mV, and 25 μM bicuculline was added to ACSF. Recording electrodes contained the following internal solution (in mM: 130 Cs-methanesulfonate, 10 CsCl, 4 NaCl, 10 HEPES, 1 MgCl_2_, 5 EGTA, 2 QX-314, 12 phosphocreatine, 5 MgATP, 0.2 Na_2_GTP, 0.1 leupeptin, pH 7.2–7.3, 265–270 mOsm).

For spontaneous inhibitory postsynaptic current (sIPSC) recording, neurons were held at −70 mV, and 25 μM CNQX was added to ACSF. The recording pipette contained the following internal solution (in mM: 100 CsCl, 30 N-methyl-Dglucamine, 10 HEPES, 4 NaCl, 1 MgCl_2_, 5 EGTA, 2 QX-314, 12 phosphocreatine, 5 MgATP, 0.5 Na_2_GTP, pH 7.2–7.3, 265–270 mOsm).

All electrophysiological recordings were performed at room temperature (21–22 °C). During recordings, neurons with leak currents >200 pA were discarded. Series resistance (Rs) was compensated to 80–90%. Cells having series resistance >10 MΩ or change above 20% throughout the experiments were excluded from analysis (Sontheimer and Ransom, 2002; Manz et al., 2021).

### 2.4 Quantitative real-time RT-PCR

Quantitative real-time PCR was performed as described in our previous studies (Ma et al., 2024a). GAPDH was used as the housekeeping gene for quantitation of the expression of target genes in samples from male and female BDNF^+/+^ and BDNF^+/Met^ mice. Fold changes in the target genes were determined by: Fold change = 2^-∆(∆CT)^, where ∆CT = C_T_ (target) - C_T_ (GAPDH), and ∆(∆C_T_) = ∆C_T_ (another group) − ∆C_T_ (male BDNF^+/+^). C_T_ (threshold cycle) is defined as the fractional cycle number at which the fluorescence reaches 10X of the standard deviation of the baseline. Primers for all target genes are listed in Table 1.

**Table 1:** List of primers used in qPCR experiments.

Target gene	Forward	Reverse	Gene reference	Length (bp)
*Gapdh*	gacaactcactcaagattgtcag	atggcatggactgtggtcatgag	NM_001289726.1	122
*Grin1*	catcggacttcagctaatca	gtccccatcctcattgaatt	NM_008169.3	238
*Grin2a*	ggctacagagacttcatcag	atccagaagaaatcgtagcc	NM_008170.4	233
*Grin2b*	ttaacaactccgtacctgtg	tggaacttcttgtcactcag	NM_008171.4	175
*Gria1*	gccttaatcgagttctgcta	gaatggattgcatggacttg	NM_008165.4	205
*Gria2*	agcctatgagatctggatgt	gagagagatcttggcgaaat	NM_001083806.3	228
*Gabra1*	caccatgaggttgaccgtga	ctacaaccactgaacgggct	NM_010250.5	158
*Gabrb2*	atttggtggctcaaacggtc	gagatttcctcaccagcagga	NM_008070.4	168
*Gabrg2*	ggagccggcatcaaatcatc	cttttggcttgtgaagcctgg	NM_008073.4	214
*Pvalb*	ggtgaagaaggtgttccata	cagacaagtctctggcatct	NM_001330686	110
*Sst*	cagactccgtcagtttctgc	atcattctctgtctggttgg	NM_009215.1	112
*Grm2*	gcttaggttcctggcact	ttaacaggtccacactcctc	NM_001160353	150
*Grm3*	caattacttgcttccaggag	tagtcaacgatgctctgaca	NM_181850	110
*Cacna1a*	ctctgctcacacttttcaca	aaaggtgatgatgatcaagg	NM_001252061.1	197
*Cacna1b*	atgctgagctgagaaaagag	ggtggttttgttctgtttgt	NM_001042528	158
*Cacna1c*	cagaaaagaaacaggaggtg	cttatcctcgttttcactgg	NM_001255999.2	152
*Cacna1d*	ggacagctctcaagatcaag	tccgtttcttgaatttccta	NM_001083616.2	204
*Cacna1e*	cgcctctattttcactcatc	catcaaggagacaaccagat	NM_009782.3	195
*Cacna2d1*	cgagacattgagaagcttct	ctggctcactctcatttctt	NM_001110843	172

### 2.5 Viral vectors and animal surgery

The AAV9-CaMKIIα-hM4D(Gi)-mCherry (Gi-GREADD) was obtained from Addgene. The virus (1 μL) was bilaterally injected into the medial PFC to infect pyramidal neurons, as we described before (Qin et al., 2019b; Qin et al., 2021). In brief, mice were anesthetized and placed on a stereotaxic apparatus (RWD Life Science, China). The injection was carried out with a Hamilton syringe (needle gauge 31) at a speed of ~0.1 μL/min, and the needle was kept in place for an additional 5 min. The virus was delivered bilaterally to the PFC using the following coordinates: 1.9 mm anterior to the bregma, 0.25 mm lateral, and 2.0 mm deep. Animals were allowed to recover for 2 days and used for experiments 2–3 weeks later. Clozapine N-oxide (CNO, 3 mg/kg) or saline via intraperitoneal injection to different BDNF^+/Met^ mice was given 1 h before the start of behavioral tests. Behavioral tests were performed at 1–6 h after intraperitoneal injection of CNO or saline in Gi-GREADD-infected mice. After behavioral tests, mice were anesthetized and perfused with PBS, followed by 4% paraformaldehyde (PFA). The brains were cut into coronal slices (80 μm) after being post-fixed in 4% PFA overnight. PFC-containing brain slices were counterstained with DAPI (nuclei). The expression of mCherry fluorescence in the PFC-containing brain slices was examined or imaged with a fluorescent microscope to confirm accurate viral targeting of the medial PFC. No sample was excluded from the analysis.

### 2.6 Social preference test

Social preference test was performed as in our previous studies (Ma et al., 2023; Qin et al., 2019b; Qin et al., 2018).

### 2.7 Statistical analysis

Data was analyzed with GraphPad Prism 10 (GraphPad) and Clampfit 11.2 (Molecular Devices, Sunnyvale, CA). For statistical significance, experiments with more than two groups were assessed with two-way or three-way ANOVA, followed by *post hoc* Bonferroni tests for multiple comparisons. All values were presented as mean ± SEM. *p* < 0.05 was considered statistically different.

## 3 Results

### 3.1 Diminished activity-dependent BDNF signaling differentially increases the frequency of spontaneous action potentials of pyramidal neurons in the PFC of male and female mice

**Figure 1:**
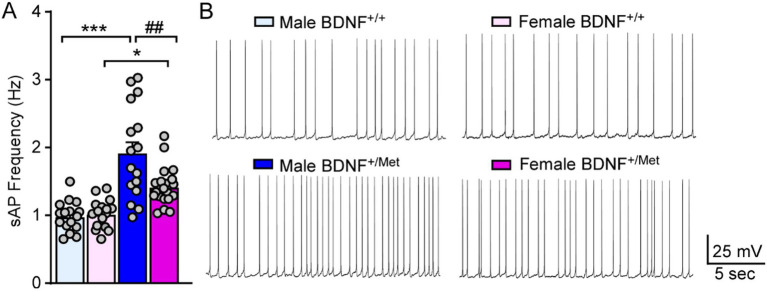
Diminished activity-dependent BDNF signaling differentially increases the frequency of spontaneous action potentials (sAPs) of pyramidal neurons in the PFC of male and female mice. Bar graphs showing the frequency **(A)** and representative traces **(B)** of sAPs in the pyramidal neurons of PFC from male and female BDNF^+/+^ and BDNF^+/Met^ mice. *p < 0.05, ***p < 0.01, BDNF^+/Met^ mice versus BDNF^+/+^ mice; ^##^*p* < 0.01, male versus female. *n* = 16–20 neurons/4–5 mice/group, two-way ANOVA.

To determine the impact of diminished activity-dependent BDNF signaling on neuronal excitability driven by synaptic inputs, the spontaneous action potential (sAP) of pyramidal neurons in the PFC of male and female BDNF^+/Met^ mice was measured by using whole-cell patch-clamp brain slice recordings. Consistent with our previous study (Ma et al., 2024a), pyramidal neurons in layer V were selected for electrophysiological measurements, since deep layer pyramidal neurons in PFC showed the clearest deficits in autistic children (Stoner et al., 2014). As shown in Figures 1A,B, diminished activity-dependent BDNF signaling significantly increased the frequency of sAPs in male and female BDNF^+/Met^ mice compared to controls. The frequency of sAP was higher in male BDNF^+/Met^ mice than in female BDNF^+/Met^ mice [male BDNF^+/+^: 0.99 ± 0.06 Hz; female BDNF^+/+^: 1.03 ± 0.05 Hz; male BDNF^+/Met^: 1.93 ± 0.17 Hz; female BDNF^+/Met^: 1.43 ± 0.06 Hz. *F*_Genotype (1, 64)_ = 48.2, *p* < 0.001; *F*_Sex (1, 64)_ = 5.8, *p* = 0.019, two-way ANOVA]. These results demonstrated that diminished activity-dependent BDNF signaling differentially increased neuronal excitability of pyramidal neurons in the PFC of male and female mice *ex vivo*, which was driven by synaptic inputs.

### 3.2 Diminished activity-dependent BDNF signaling differentially regulates excitatory and inhibitory synaptic transmission of pyramidal neurons in the PFC of male and female mice

**Figure 2:**
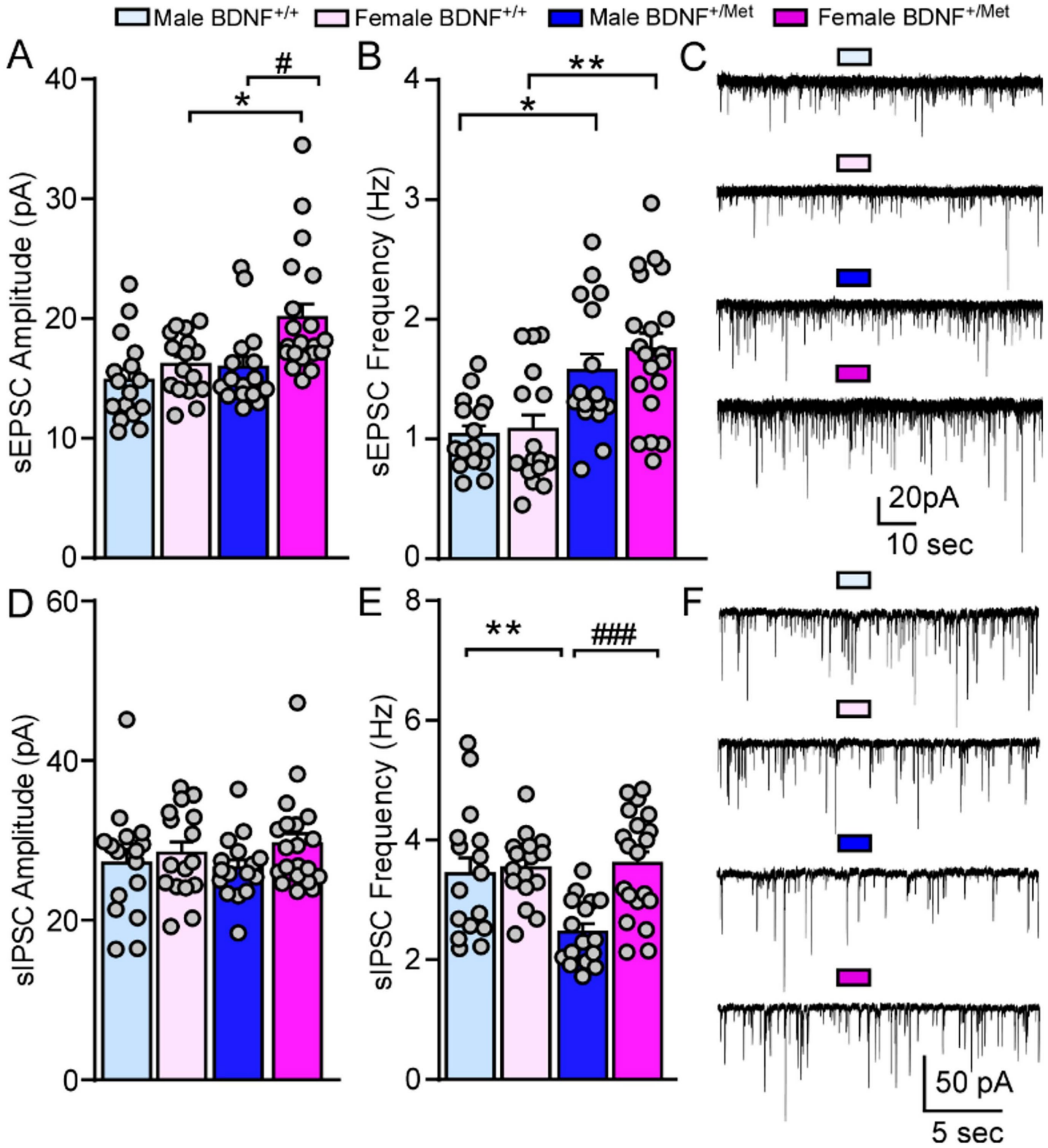
Diminished activity-dependent BDNF signaling differentially regulates excitatory and inhibitory synaptic transmission of pyramidal neurons in the PFC of male and female mice. Bar graphs showing the amplitude **(A)** and the frequency **(B)** of sEPSC in the pyramidal neurons of PFC from male and female BDNF^+/+^ and BDNF^+/Met^ mice. **(C)** Representative traces of sEPSC. Bar graphs showing the amplitude **(D)** and the frequency **(E)** of sIPSC in the pyramidal neurons of PFC from male and female BDNF^+/+^ and BDNF^+/Met^ mice. **(F)** Representative traces of sIPSC. *p < 0.05, **p < 0.01, BDNF^+/Met^ mice versus BDNF^+/+^ mice; ^#^*p* < 0.05, ^###^*p* < 0.001, male versus female. *n* = 16–20 neurons/4–5 mice/group, two-way ANOVA.

To find out the physiological basis of the differential hyperexcitability of pyramidal neurons in male and female BDNF^+/Met^ mice, we further examined excitatory and inhibitory synaptic transmission by measuring AMPA (*α*-amino-3-hydroxy-5-methyl-4-isoxazolepropionic acid) receptor-mediated spontaneous excitatory postsynaptic currents (sEPSC) and GABA_A_ (*γ*-aminobutyric acid) receptor-mediated spontaneous inhibitory postsynaptic currents (sIPSC). As shown in Figures 2A–F, diminished activity-dependent BDNF significantly increased the amplitude of sEPSC in female mice but not in male mice [amplitude: male BDNF^+/+^: 14.85 ± 0.89 pA; female BDNF^+/+^: 16.19 ± 0.64 pA; male BDNF^+/Met^: 15.95 ± 0.86 pA; female BDNF^+/Met^: 20.07 ± 1.15 pA. *F*_Genotype (1, 64)_ = 6.90, *p* = 0.011; *F*_Sex (1, 64)_ = 8.32, *p* = 0.005, two-way ANOVA]. The frequencies of sEPSC were similarly increased in male and female BDNF^+/Met^ mice compared to male and female BDNF^+/+^ mice [frequency: male BDNF^+/+^: 1.04 ± 0.07 Hz; female BDNF^+/+^: 1.08 ± 0.12 Hz; male BDNF^+/Met^: 1.57 ± 0.14 Hz; female BDNF^+/Met^: 1.75 ± 0.13 Hz. *F*_Genotype (1, 64)_ = 24.08, *p* < 0.001; *F*_Sex (1, 64)_ = 0.8, *p* = 0.37, two-way ANOVA]. Diminished activity-dependent BDNF signaling had no effect on the amplitudes of sIPSC in males and females [amplitude: male BDNF^+/+^: 27.19 ± 1.5 pA; female BDNF^+/+^: 28.44 ± 1.39 pA; male BDNF^+/Met^: 26.60 ± 0.99 pA; female BDNF^+/Met^: 29.57 ± 1.29 pA. *F*_Genotype (1, 64)_ = 0.04, *p* = 0.85; *F*_Sex (1, 64)_ = 2.33, *p* = 0.13, two-way ANOVA]. However, diminished activity-dependent BDNF signaling significantly decreased the frequency of sIPSC in males but not in females [frequency: male BDNF^+/+^: 3.44 ± 0.27 Hz; female BDNF^+/+^: 3.54 ± 0.15 Hz; male BDNF^+/Met^: 2.47 ± 0.14 Hz; female BDNF^+/Met^: 3.61 ± 0.2 Hz. *F*_Genotype (1, 64)_ = 5.27, *p* = 0.025; *F*_Sex (1, 64)_ = 10.01, *p* = 0.002, two-way ANOVA]. These results demonstrated that diminished activity-dependent BDNF dampened presynaptic GABAergic inhibition and enhanced presynaptic glutamatergic excitation in males and increased pre- and post-synaptic glutamatergic excitation in females, which differentially increased excitability of pyramidal neurons in males and females.

### 3.3 Diminished activity-dependent BDNF signaling differentially regulates the transcriptional levels of key synaptic genes in the PFC of male and female mice

**Figure 3:**
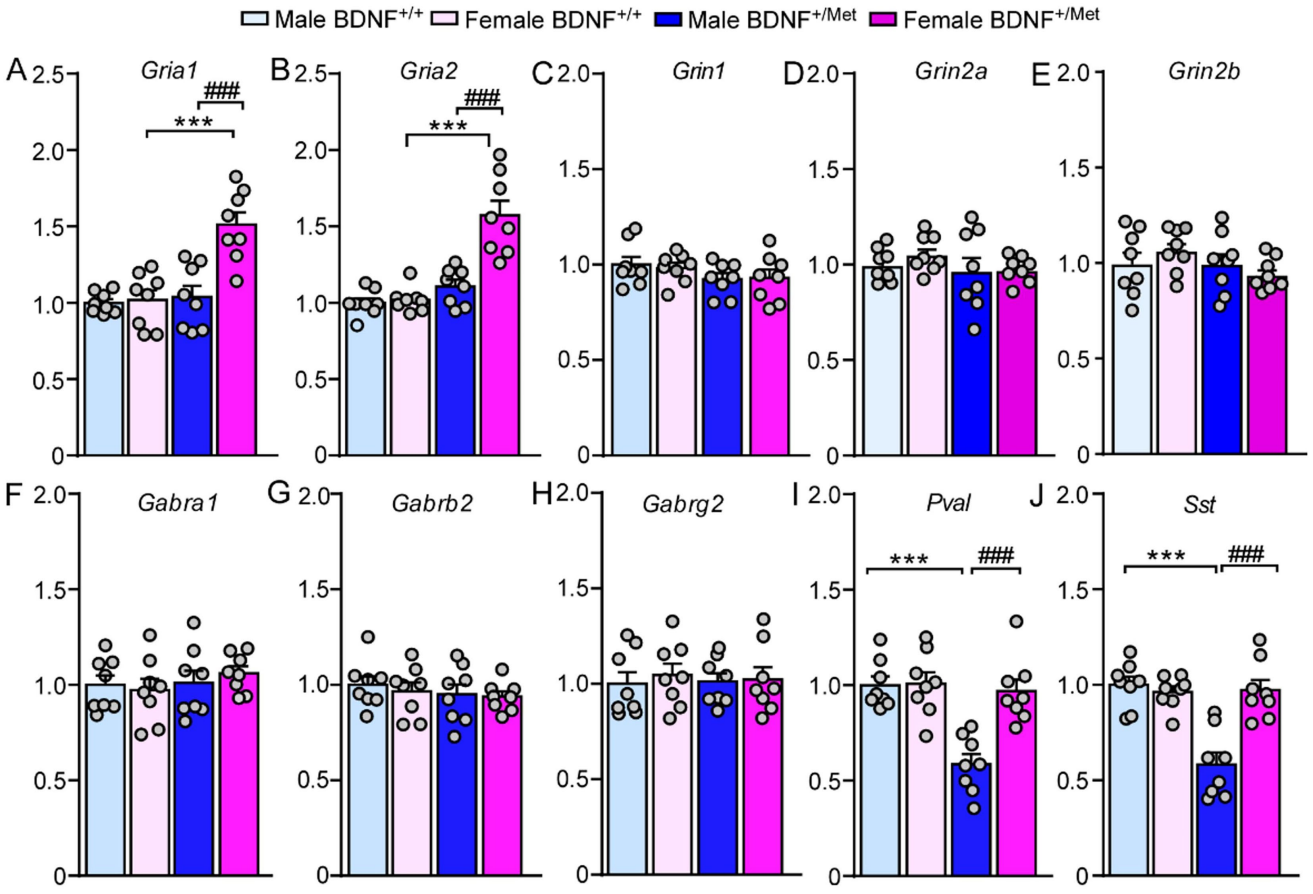
Diminished activity-dependent BDNF signaling differentially regulates the key excitatory and inhibitory synaptic genes in the PFC of male and female mice. Quantitative real-time PCR showing the transcriptional level of the key excitatory and inhibitory synaptic genes in the PFC of male and female BDNF^+/+^ and BDNF^+/Met^ mice. **(A–E)** Excitatory synaptic genes. **(F–J)** Inhibitory synaptic genes. ***p < 0.001, BDNF^+/Met^ mice versus BDNF^+/+^ mice; ^###^*p* < 0.001, male versus female. *n* = 8 mice/group, two-way ANOVA.

To determine the molecular mechanisms underlying the imbalance of excitatory and inhibitory synaptic transmission in male and female BDNF^+/Met^ mice, the transcriptional levels of the key excitatory and inhibitory synaptic genes in the PFC were examined by real-time PCR. As shown in Figure 3, the mRNA levels of *Gria1/2* (encoding GluR1/2, key subunits of AMPA receptors) were significantly increased in female BDNF^+/Met^ mice [*Gria1*: male BDNF^+/+^: 1.0 ± 0.02; female BDNF^+/+^: 1.02 ± 0.06; male BDNF^+/Met^: 1.04 ± 0.07; female BDNF^+/Met^: 1.51 ± 0.08. *F*_Genotype (1, 28)_ = 16.84*, p* < 0.001; *F*_sex (1, 28)_ = 14.52*, p* < 0.001; *Gria2*: male BDNF^+/+^: 1.0 ± 0.03; female BDNF^+/+^:1.02 ± 0.03; male BDNF^+/Met^: 1.11 ± 0.04; female BDNF^+/Met^: 1.57 ± 0.09. *F*_Genotype (1, 28)_ = 35.93*, p* < 0.001; *F*_sex (1, 28)_ = 19.43*, p* = 0.001, two-way ANOVA], while the mRNA levels of *Grin1* (encoding NR1, key subunit of N-methyl-D-aspartate, NMDA receptors), *Grin2a/b* (encoding NR2A/B, key subunits of NMDA receptors) were largely unchanged [*Grin1*: male BDNF^+/+^: 1.0 ± 0.04; female BDNF^+/+^: 0.98 ± 0.03; male BDNF^+/Met^: 0.92 ± 0.03; female BDNF^+/Met^: 0.93 ± 0.04. *F*_Genotype (1, 28)_ = 3.17*, p* = 0.08; *F*_sex (1, 28)_ = 0.02*, p* = 0.88; *Grin2a*: male BDNF^+/+^: 1.0 ± 0.03; female BDNF^+/+^: 1.06 ± 0.03; male BDNF^+/Met^: 0.97 ± 0.07; female BDNF^+/Met^: 0.97 ± 0.02. *F*_Genotype (1, 28)_ = 1.48*, p* = 0.23; *F*_sex (1, 28)_ = 0.44*, p* = 0.51; *Grin2b*: male BDNF^+/+^: 1.0 ± 0.06; female BDNF^+/+^: 1.07 ± 0.04; male BDNF^+/Met^: 0.99 ± 0.06; female BDNF^+/Met^: 0.94 ± 0.03. *F*_Genotype (1, 28)_ = 1.69*, p* = 0.20; *F*_sex (1, 28)_ = 0.01*, p* = 0.91, two-way ANOVA]. The inhibitory synaptic genes *Gabra1/b2/g2* (encoding GABA_A_ receptor α1, β2, and γ2 subunits) were not changed [*Gabra1*: male BDNF^+/+^: 1.0 ± 0.05; female BDNF^+/+^: 0.97 ± 0.06; male BDNF^+/Met^: 1.01 ± 0.06; female BDNF^+/Met^: 1.06 ± 0.04. *F*_Genotype (1, 28)_ = 0.89*, p* = 0.35; *F*_sex (1, 28)_ = 0.04*, p* = 0.83; *Gabrb2*: male BDNF^+/+^: 1.0 ± 0.04; female BDNF^+/+^: 0.97 ± 0.05; male BDNF^+/Met^: 0.95 ± 0.05; female BDNF^+/Met^: 0.94 ± 0.03. *F*_Genotype (1, 28)_ = 0.79*, p* = 0.38; *F*_sex (1, 28)_ = 0.27*, p* = 0.61; *Gabrg2*: male BDNF^+/+^: 1.0 ± 0.06; female BDNF^+/+^: 1.05 ± 0.06; male BDNF^+/Met^: 1.01 ± 0.04; female BDNF^+/Met^: 1.02 ± 0.06. *F*_Genotype (1, 28)_ = 0.01*, p* = 0.93; *F*_sex (1, 28)_ = 0.25*, p* = 0.62, two-way ANOVA], while *Pvalb* (encoding Parvalbumin) and *Sst* (encoding Somatostatin) were significantly decreased in male BDNF^+/Met^ mice [*Pvalb*: male BDNF^+/+^: 1.0 ± 0.05; female BDNF^+/+^: 1.01 ± 0.06; male BDNF^+/Met^: 0.59 ± 0.05; female BDNF^+/Met^: 0.97 ± 0.06. *F*_Genotype (1, 28)_ = 17.14*, p* < 0.001; *F*_sex (1, 28)_ = 12.74*, p* = 0.0013; *Sst*: male BDNF^+/+^: 1.0 ± 0.04; female BDNF^+/+^: 0.96 ± 0.03; male BDNF^+/Met^: 0.58 ± 0.06; female BDNF^+/Met^: 0.97 ± 0.05. *F*_Genotype (1, 28)_ = 17.45*, p* = 0.0003; *F*_sex (1, 28)_ = 13.01*, p* = 0.0012, two-way ANOVA].

**Figure 4:**
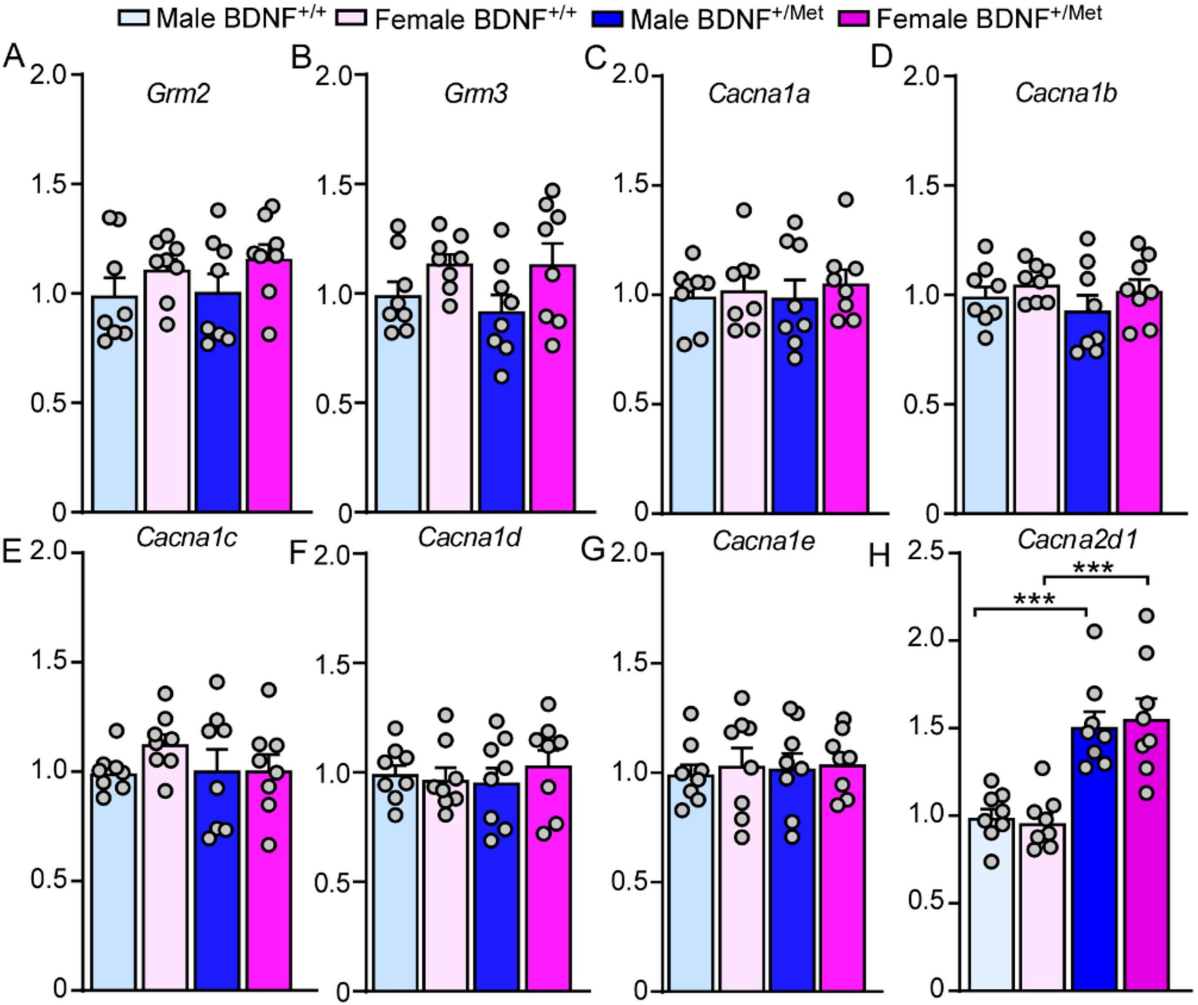
The impact of diminished activity-dependent BDNF signaling on the expressions of key molecules involving presynaptic glutamate releases in the PFC of male and female mice. (A-H) Quantitative real-time PCR showing the transcriptional level of the key molecules involving presynaptic glutamate releases in the PFC of male and female BDNF^+/+^ and BDNF^+/Met^ mice. ***p < 0.001, BDNF^+/Met^ mice versus BDNF^+/+^ mice. *n* = 8 mice/group, two-way ANOVA.

To determine the potential mechanisms for the increased frequencies of sEPSC in male and female BDNF^+/Met^ mice, the key molecules that mediate the presynaptic releases of glutamate were examined. As shown in Figure 4, the mRNA levels of *Grm2/3* (encoding mGluR2/3) were not changed in the PFC of male and female BDNF^+/Met^ mice compared to male and female BDNF^+/+^ mice [*Grm2*: male BDNF^+/+^: 1.0 ± 0.08; female BDNF^+/+^: 1.12 ± 0.05; male BDNF^+/Met^: 1.02 ± 0.08; female BDNF^+/Met^: 1.17 ± 0.07. *F*_Genotype (1, 28)_ = 0.21*, p* = 0.65; *F*_sex (1, 28)_ = 3.44*, p* = 0.07; *Grm3*: male BDNF^+/+^: 1.0 ± 0.06; female BDNF^+/+^: 1.15 ± 0.04; male BDNF^+/Met^: 0.93 ± 0.08; female BDNF^+/Met^: 1.14 ± 0.09. *F*_Genotype (1, 28)_ = 0.27*, p* = 0.61; *F*_sex (1, 28)_ = 6.10*, p* = 0.02. *Post-hoc* Bonferroni’s multiple comparisons test: Adjusted *p* > 1.0 for male BDNF^+/+^ versus female BDNF^+/+^ mice; Adjust *p* = 0.28 for male BDNF^+/Met^ versus female BDNF^+/Met^ mice, two-way ANOVA]. The mRNA levels of *Cacna2d1* (encoding α2δ1 subunit of high- and intermediate-voltage activated calcium channels) were similarly higher in male and female BDNF^+/Met^ mice than in male and female BDNF^+/+^ mice [*Cacna2d1*: male BDNF^+/+^: 1.0 ± 0.05; female BDNF^+/+^: 0.97 ± 0.05; male BDNF^+/Met^: 1.52 ± 0.09; female BDNF^+/Met^: 1.56 ± 0.12. *F*_Genotype (1, 28)_ = 44.6, *p* < 0.001; *F*_Sex (1, 28)_ = 0.005, *p* = 0.95, two-way ANOVA], while the mRNA levels of *Cacna1a* (encoding α1A subunit of Cav 2.1), *Cacna1b* (encoding α1B subunit of Cav 2.2), *Cacna1c* (encoding α1C subunit of Cav 1.2), *Cacna1d* (encoding α1D subunit of Cav 1.3), and *Cacna1e* (encoding Cav 2.3) were largely unchanged [*Cacna1a*: male BDNF^+/+^: 1.0 ± 0.05; female BDNF^+/+^: 1.03 ± 0.07; male BDNF^+/Met^: 1.00 ± 0.08; female BDNF^+/Met^: 1.06 ± 0.06. *F*_Genotype (1, 28)_ = 0.04*, p* = 0.84; *F*_sex (1, 28)_ = 0.51*, p* = 0.48; *Cacna1b*: male BDNF^+/+^: 1.0 ± 0.05; female BDNF^+/+^: 1.06 ± 0.03; male BDNF^+/Met^: 0.94 ± 0.07; female BDNF^+/Met^: 1.03 ± 0.05. *F*_Genotype (1, 28)_ = 0.73*, p* = 0.39; *F*_sex (1, 28)_ = 1.93*, p* = 0.18; *Cacna1c*: male BDNF^+/+^: 1.0 ± 0.03; female BDNF^+/+^: 1.13 ± 0.05; male BDNF^+/Met^: 1.02 ± 0.09; female BDNF^+/Met^: 1.01 ± 0.07. *F*_Genotype (1, 28)_ = 0.60*, p* = 0.45; *F*_sex (1, 28)_ = 0.97*, p* = 0.33; *Cacna1d*: male BDNF^+/+^: 1.0 ± 0.05; female BDNF^+/+^: 0.97 ± 0.05; male BDNF^+/Met^: 0.96 ± 0.07; female BDNF^+/Met^: 1.04 ± 0.07. *F*_Genotype (1, 28)_ = 0.06*, p* = 0.81; *F*_sex (1, 28)_ = 0.17*, p* = 0.68; *Cacna1e*: male BDNF^+/+^: 1.0 ± 0.05; female BDNF^+/+^: 1.04 ± 0.08; male BDNF^+/Met^: 1.03 ± 0.07; female BDNF^+/Met^: 1.05 ± 0.05. *F*_Genotype (1, 28)_ = 0.07*, p* = 0.079; *F*_sex (1, 28)_ = 0.21*, p* = 0.65, two-way ANOVA]. These results suggested that the increased expression of *Cacna2d1* may enhance the membrane density or alter the biophysical properties of voltage-gated Ca^2+^ channels, facilitating glutamate release (Dolphin, 2018), which contributed to the increased frequency of sEPSC in male and female BDNF^+/Met^ mice.

### 3.4 Chemogenetic inhibition of pyramidal neurons in the PFC ameliorates autism-like social preference deficits in male and female BDNF^+/Met^ mice

**Figure 5:**
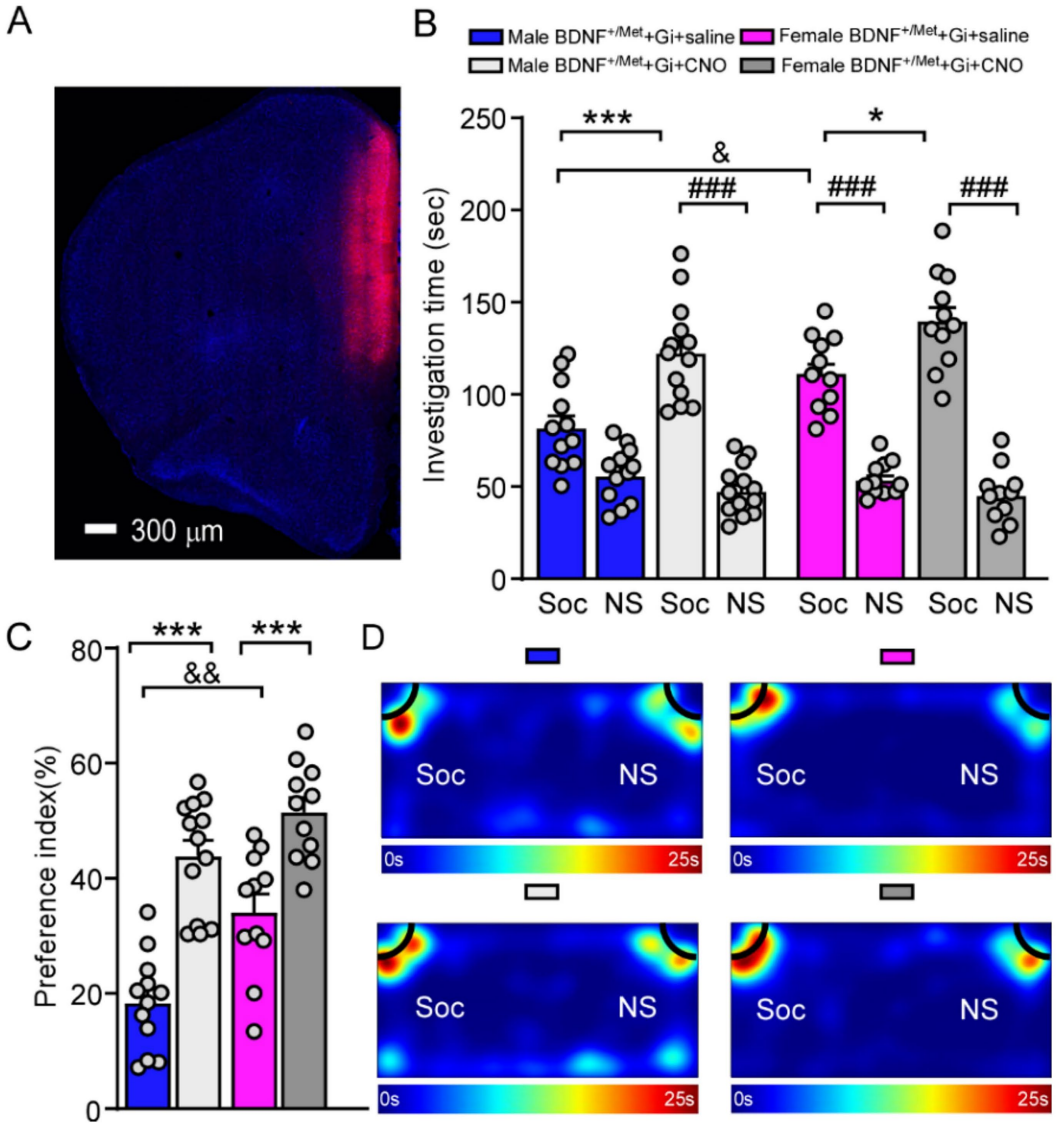
Chemogenetic inhibition of PFC ameliorates autism-like social preference deficits in male and female BDNF^+/Met^ mice. **(A)** A confocal image showing the expression of Gi-DREADD in the medial PFC (stained with DAPI, blue) from a BDNF^+/Met^ mouse. Scale bar: 300 μm. Bar graphs showing the time spent investigating social (Soc) and non-social (NS) stimulus **(B)** and social preference index **(C)** in the three-chamber sociability test of BDNF^+/Met^ mice (Gi-DREADD) treated with saline or CNO. ^###^*p* < 0.001, Soc versus NS; *p < 0.05, ***p < 0.001, CNO versus saline; ^&^*p* < 0.05, ^&&^*p* < 0.01, male versus female. **(D)** Representative heatmaps illustrating the time spent in different locations from the social preference tests. Male BDNF^+/Met^ + Gi + saline: *n* = 12 mice; Male BDNF^+/Met^ + Gi + CNO: *n* = 13 mice; Female BDNF^+/Met^ +Gi + saline: *n* = 11 mice; Female BDNF^+/Met^ + Gi + CNO: *n* = 11 mice. **(B)** Three-way ANOVA. **(C)** Two-way ANOVA.

Designer receptors exclusively activated by designer drugs (DREADDs) is a powerful chemogenetic technology for remote activation or inhibition of neuronal activity (Rogan and Roth, 2011; Roth, 2016; Armbruster et al., 2007; Zhang et al., 2022). To determine if inhibition of pyramidal neurons in the PFC could rescue social preference deficits in male and female BDNF^+/Met^ mice, we injected mCherry-tagged CaMKII-driven Gi-DREADD adeno-associated virus (AAV) bilaterally into the medial PFC, which facilitates Gi-DREADD expression in the pyramidal neurons (Figure 5A). As shown in Figures 5B-D, in the three-chamber sociability assay, CNO (3 mg/kg, i.p.)-treated male and female BDNF^+/Met^ mice (hM4Di-injected) spent significantly more time exploring the social stimulus over the non-social object, whereas saline-treated male and female BDNF^+/Met^ mice showed a significant loss of the preference for the social stimulus [male BDNF^+/Met^ + Gi + saline: social time: 82.6 ± 6.7 s, nonsocial time: 56.7 ± 4.3 s; male BDNF^+/Met^ + Gi + CNO: social time: 123.3 ± 7.5 s, nonsocial time: 48.2 ± 3.8 s; female BDNF^+/Met^ + Gi + saline: social time: 112.1 ± 6.2 s, nonsocial time: 54.4 ± 2.8 s; female BDNF^+/Met^ + Gi + CNO: social time: 140.7 ± 8.0 s, nonsocial time: 45.9 ± 4.5 s; *F*_Treatment (1, 86)_ = 10.3, *p* = 0.0019; *F*_Soc vs. NS (1, 86)_ = 241.1, *p* < 0.001; *F*_Sex (1, 86)_ = 6.7, *p* = 0.0113, three-way ANOVA]. The social preference index was significantly elevated in male and female BDNF^+/Met^ mice after CNO treatment [male BDNF^+/Met^ + Gi + saline: 18.5% ± 2.4%; male BDNF^+/Met^ + Gi + CNO: 43.9% ± 2.8%; female BDNF^+/Met^ + Gi + saline: 34.2% ± 3.2%; female BDNF^+/Met^ + Gi + CNO: 51.6% ± 2.6%; *F*_Treatment (1, 43)_ = 60.6, *p* < 0.001; *F*_Sex (1, 43)_ = 18.1, *p* = 0.0001, two-way ANOVA], indicating amelioration of autism-like social preference deficits. These results demonstrated that diminished activity-dependent BDNF signaling-induced social preference deficits in male and female mice could be mitigated by inhibition of pyramidal neuronal activity in the PFC.

## 4 Discussion

To decipher the potential neural mechanisms underlying the differential severity of social preference deficits between male and female BDNF^+/Met^ mice (Ma et al., 2023), in this study, we characterized the impact of diminished activity-dependent BDNF signaling on the synaptic transmission of pyramidal neurons in the PFC of male and female mice. Diminished activity-dependent BDNF signaling differentially increased the frequency of sAPs in the pyramidal neurons of PFC in male and female mice. Furthermore, diminished activity-dependent BDNF signaling differentially regulated excitatory and inhibitory synaptic transmission in males and females. The sex differences of diminished activity-dependent BDNF signaling on the intrinsic excitability (Ma et al., 2024a), excitatory and inhibitory synaptic transmission of pyramidal neurons in the PFC potentially contributed to the differential severity of social preference deficits in male and female BDNF^+/Met^ mice, which were ameliorated by chemogenetic inhibition.

Vesicular glutamate transporter 1/2 (VGLUT1/2) are specific markers for excitatory glutamatergic neurons, which load glutamate into presynaptic vesicles (Bellocchio et al., 2000; Moechars et al., 2006). The transcriptional levels of VGLUT1/2 were increased in male BDNF^+/Met^ mice with unknown changes in female BDNF^+/Met^ mice (Qin et al., 2014). The increased frequency of sEPSC in the pyramidal neurons of male and female BDNF^+/Met^ mice indicated that the presynaptic glutamate releases were enhanced. The metabotropic glutamate receptors 2 and 3 (mGluR2/3) are group II G protein-coupled receptors that inhibit excess glutamate release (Celli et al., 2019; Benneyworth et al., 2007). The diminished activity-dependent BDNF signaling had no effects on the mRNA levels of *Grm2/3* in males and females, which indicated that the mGluR2/3 did not contribute to the increased releases of glutamate in male and female BDNF^+/Met^ mice. Voltage-gated calcium channels (VGCCs) contribute to gene transcription and presynaptic glutamate releases, which are composed of a principal pore-forming α1 subunit and several auxiliary subunits (Lai and Jan, 2006; Dolphin, 2016). The transcriptional levels of *Cacna1b/c* were significantly upregulated in the hippocampal CA3 pyramidal neurons of female BDNF^+/Met^ and downregulated in male BDNF^+/Met^ (Marrocco et al., 2017). In the PFC, the expressions of key α1 subunits of VGCCs were not changed in male and female BDNF^+/Met^ mice compared to male and female BDNF^+/+^ mice. *Cncna2d1* encodes α2δ1, one of the key auxiliary subunits of VGCCs that influences the channels’ biophysical properties (Dolphin, 2012) and plays critical roles in excitatory synaptogenesis (Eroglu et al., 2009). The transcriptional level of *Cncna2d1* was selectively upregulated in the PFC of male and female BDNF^+/Met^ mice, which is consistent with our previous studies (Qin et al., 2019a). Cav 2.2, an N-type α2δ1-containing VGCC, is critical for presynaptic neurotransmitter release (Jurkovicova-Tarabova and Lacinova, 2019; Heyes et al., 2015). Current transcriptional and electrophysiological data suggested that Cav 2.2 potentially contributed to the increased frequency of sEPSC in pyramidal neurons of male and female BDNF^+/Met^ mice. Further functional studies are needed to confirm that the increased α2δ1 increases the membrane density or alters the biophysical properties of Cav 2.2 channels, facilitating glutamate release (Dolphin, 2018).

AMPA receptors and NMDA receptors are key ionotropic glutamate receptors, which are located on the pyramidal neurons (Rotaru et al., 2011; Wang et al., 2008). Significant reductions of mRNA levels of key subunits of AMPA receptors and NMDA receptors were found in autistic patients (Qin et al., 2021). *SHANK3* (SH3 and multiple ankyrin repeat domains 3) is a prominent gene for ASD (Satterstrom et al., 2020; De Rubeis et al., 2014), which is a scaffolding protein located at postsynaptic density (PSD) of excitatory synapses. Diminished NMDA receptor- and/or AMPA receptor-mediated excitatory synaptic transmission contributed to the social deficits in *Shank3* haploinsufficiency mouse models (Qin et al., 2018; Duffney et al., 2015; Wang X. et al., 2016). The increased mRNA levels of *Gria1/2* but not *Grin1* and *Grin2a/b* indicated that AMPA receptors contributed to the increased amplitudes of sEPSC in pyramidal neurons of female BDNF^+/Met^ mice.

The decreased frequency of sIPSC in the pyramidal neurons of male BDNF^+/Met^ mice indicated that the presynaptic GABA releases were dampened. In the cortex, about 40% of interneurons are parvalbumin (PV^+^)-expressing fast-spiking interneurons, including basket cells and chandelier cells, and about 30% of interneurons are somatostatin (SST^+^)-expressing interneurons (Tremblay et al., 2016; Druga, 2009). PV^+^ interneurons possessing high-frequency firing rates electrophysiological properties are critical for regulating synaptic E/I balance, which constitutes the dominant cortical inhibitory cell type (Rudy et al., 2011). PV^+^ basket cells target the soma and proximal dendrites of pyramidal neurons, inhibiting pyramidal neuronal excitability. PV^+^ chandelier cells project on the axonal initial segment of pyramidal neurons, thus modulating the output spike generation and spike timing of pyramidal neurons (Jiang et al., 2016). SST^+^ interneurons synapse on the dendrites of pyramidal neurons, where they refine the glutamatergic inputs on cortical pyramidal neurons and modulate the dendritic synaptic integration and plasticity of pyramidal neurons (Lovett-Barron et al., 2012). Decreased numbers of PV^+^ neurons were found in the PFC from postmortem brain tissue of autism patients (Hashemi et al., 2017). Histone methyltransferase *ASH1L* is a high-risk gene for ASD (Satterstrom et al., 2020; De Rubeis et al., 2014; Faundes et al., 2018; Stessman et al., 2017). *Ash1l* haploinsufficiency significantly downregulated the expressions of *Pval* (encoding PV) and *Sst* (encoding SST) in the PFC of mice (Ma et al., 2024b). The decreased mRNA levels of *Pval* and *Sst* in the PFC of male BDNF^+/Met^ mice indicated their key roles in the decreased frequency of sIPSC in the pyramidal neurons of male BDNF^+/Met^ mice. The unaltered mRNA levels of key subunits of GABA_A_ receptors in male and female BDNF^+/Met^ mice demonstrated that diminished activity-dependent BDNF signaling had no effects on the inhibitory synaptic transmission at postsynaptic levels. Consistently, there was a sex dimorphic expression of excitatory and inhibitory transmission of CA3 pyramidal neurons in the hippocampus of male and female BDNF^+/Met^ mice (Marrocco et al., 2017). However, the sex-effects of diminished activity-dependent BDNF signaling on the excitatory and inhibitory balance in the PFC and CA3 are in a region-specific manner.

There are some limitations in this study. First, the mechanisms of how chemogenetic inhibition of pyramidal neurons in the PFC ameliorated autism-like social preference deficits in male and female BDNF^+/Met^ are unclear. A resting-state functional magnetic resonance imaging (fMRI) brain study from ASD patients showed hypoconnectivity in the brains of males and hyperconnectivity in females, which was potentially driven by sex hormonal alterations (Alaerts et al., 2016). Sex hormones influence brain structure and neural circuits that mediate social behaviors (Gegenhuber et al., 2022). PFC is reciprocally connected with subcortical regions (Murugan et al., 2017; Zhong et al., 2022; Zhong et al., 2020). The potential sex-specific neural circuits and circuit-specific molecular mechanisms underlying the chemogenetic restoration of social preference deficits in male and female BDNF^+/Met^ mice need to be further investigated. Second, the medial PFC is mainly composed of infralimbic cortex and prelimbic prefrontal cortex, which are both included in this study. It has been reported that the infralimbic cortex and prelimbic prefrontal cortex play distinct roles in social behaviors (Sato et al., 2023). Further studies are needed to clarify whether the infralimbic cortex and prelimbic prefrontal cortex are differentially affected in male and female BDNF^+/Met^ mice.

In summary, the present study revealed synaptic mechanisms mediated the differential severity of social preference deficits in male and female BDNF^+/Met^ mice (Ma et al., 2023). In combination with our previous findings that increased intrinsic excitability of pyramidal neurons in male BDNF^+/Met^ mice but not in female BDNF^+/Met^ mice (Ma et al., 2024a), these results provided a possible neural basis underpinning that males are more vulnerable to having ASD (Giarelli et al., 2010; Werling and Geschwind, 2013). Carbamoylated erythropoietin (cEPO), a chemically modified erythropoietin, could increase the transcriptional level of BDNF in the PFC without showing hematological activity (Sampath et al., 2020; Sathyanesan et al., 2018). cEPO treatment could rescue autism-relevant social deficits in BALB/cJ mice (Street et al., 2025). To support that diminished activity-dependent neural signaling is a common pathway in ASD and to develop mechanism-based therapeutic strategies for ASD patients, further studies to examine the therapeutic effects of cEPO on the social preference deficits in male and female BDNF^+/Met^ mice are required.

## Data Availability

The raw data supporting the conclusions of this article will be made available by the authors, without undue reservation.

## Author’s note

All authors confirm that the following manuscript is a transparent and honest account of the reported research. This research is related to our previous studies titled “Diminished activity-dependent BDNF signaling differentially causes autism-like behavioral deficits in male and female mice” and “A sexually dimorphic signature of activity-dependent BDNF signaling on the intrinsic excitability of pyramidal neurons in the prefrontal cortex.” The previous studies examined the impact of diminished activity-dependent BDNF signaling on the autism-like behaviors and intrinsic excitability of pyramidal neurons in the PFC of male and female mice, and the current submission focuses on the synaptic mechanisms for diminished activity-dependent BDNF-induced differential severity of social preference deficits in males and females. The study is following the methodology explained in Frontiers in Psychiatry. 2023;14:1182472 and Front Cell Neurosci. 2024;18: 1496930.

## References

[ref1] Alaerts K. Swinnen S. P. Wenderoth N. (2016). Sex differences in autism: a resting-state fMRI investigation of functional brain connectivity in males and females. Soc. Cogn. Affect. Neurosci. 11, 1002–1016. doi: 10.1093/scan/nsw027, 26989195 PMC4884321

[ref2] Armbruster B. N. Li X. Pausch M. H. Herlitze S. Roth B. L. (2007). Evolving the lock to fit the key to create a family of G protein-coupled receptors potently activated by an inert ligand. Proc. Natl. Acad. Sci. USA 104, 5163–5168. doi: 10.1073/pnas.0700293104, 17360345 PMC1829280

[ref3] Arnsten A. F. Rubia K. (2012). Neurobiological circuits regulating attention, cognitive control, motivation, and emotion: disruptions in neurodevelopmental psychiatric disorders. J. Am. Acad. Child Adolesc. Psychiatry 51, 356–367. doi: 10.1016/j.jaac.2012.01.008, 22449642

[ref4] Bellocchio E. E. Reimer R. J. Fremeau R. T. Edwards R. H. (2000). Uptake of glutamate into synaptic vesicles by an inorganic phosphate transporter. Science 289, 957–960. doi: 10.1126/science.289.5481.957, 10938000

[ref5] Benneyworth M. A. Xiang Z. Smith R. L. Garcia E. E. Conn P. J. Sanders-Bush E. (2007). A selective positive allosteric modulator of metabotropic glutamate receptor subtype 2 blocks a hallucinogenic drug model of psychosis. Mol. Pharmacol. 72, 477–484. doi: 10.1124/mol.107.035170, 17526600

[ref6] Celli R. Santolini I. Van Luijtelaar G. Ngomba R. T. Bruno V. Nicoletti F. (2019). Targeting metabotropic glutamate receptors in the treatment of epilepsy: rationale and current status. Expert Opin. Ther. Targets 23, 341–351. doi: 10.1080/14728222.2019.1586885, 30801204

[ref7] Chao M. V. (2003). Neurotrophins and their receptors: a convergence point for many signalling pathways. Nat. Rev. Neurosci. 4, 299–309. doi: 10.1038/nrn1078, 12671646

[ref8] Chen Z. Y. Jing D. Bath K. G. Ieraci A. Khan T. Siao C. J. . (2006). Genetic variant BDNF (Val66Met) polymorphism alters anxiety-related behavior. Science 314, 140–143. doi: 10.1126/science.1129663, 17023662 PMC1880880

[ref9] Davidson R. J. (2002). Anxiety and affective style: role of prefrontal cortex and amygdala. Biol. Psychiatry 51, 68–80. doi: 10.1016/S0006-3223(01)01328-2, 11801232

[ref10] De Rubeis S. He X. Goldberg A. P. Poultney C. S. Samocha K. Cicek A. E. . (2014). Synaptic, transcriptional and chromatin genes disrupted in autism. Nature 515, 209–215. doi: 10.1038/nature13772, 25363760 PMC4402723

[ref11] Dolphin A. C. (2012). Calcium channel auxiliary α2δ and β subunits: trafficking and one step beyond. Nat. Rev. Neurosci. 13, 542–555. doi: 10.1038/nrn3311, 22805911

[ref12] Dolphin A. C. (2016). Voltage-gated calcium channels and their auxiliary subunits: physiology and pathophysiology and pharmacology. J. Physiol. 594, 5369–5390. doi: 10.1113/JP272262, 27273705 PMC5043047

[ref13] Dolphin A. C. (2018). Voltage-gated calcium channel α (2)δ subunits: an assessment of proposed novel roles. F1000Res 7:1830. doi: 10.12688/f1000research.16104.1 PMC6249638 30519455

[ref14] Druga R. (2009). Neocortical inhibitory system. Folia Biol 55, 201–217. doi: 10.14712/fb2009055060201, 20163769

[ref15] Duffney L. J. Zhong P. Wei J. Matas E. Cheng J. Qin L. . (2015). Autism-like deficits in Shank3-deficient mice are rescued by targeting actin regulators. Cell Rep. 11, 1400–1413. doi: 10.1016/j.celrep.2015.04.064, 26027926 PMC4464902

[ref16] Ebert D. H. Greenberg M. E. (2013). Activity-dependent neuronal signalling and autism spectrum disorder. Nature 493, 327–337. doi: 10.1038/nature11860, 23325215 PMC3576027

[ref17] Egan M. F. Kojima M. Callicott J. H. Goldberg T. E. Kolachana B. S. Bertolino A. . (2003). The BDNF val66met polymorphism affects activity-dependent secretion of BDNF and human memory and hippocampal function. Cell 112, 257–269. doi: 10.1016/S0092-8674(03)00035-7, 12553913

[ref18] Eroglu C. Allen N. J. Susman M. W. O'Rourke N. A. Park C. Y. Ozkan E. . (2009). Gabapentin receptor alpha2delta-1 is a neuronal thrombospondin receptor responsible for excitatory CNS synaptogenesis. Cell 139, 380–392. doi: 10.1016/j.cell.2009.09.025 19818485 PMC2791798

[ref19] Faber E. S. Sah P. (2007). Functions of SK channels in central neurons. Clin. Exp. Pharmacol. Physiol. 34, 1077–1083. doi: 10.1111/j.1440-1681.2007.04725.x, 17714097

[ref20] Faundes V. Newman W. G. Bernardini L. Canham N. Clayton-Smith J. Dallapiccola B. . (2018). Histone lysine methylases and demethylases in the landscape of human developmental disorders. Am. J. Hum. Genet. 102, 175–187. doi: 10.1016/j.ajhg.2017.11.013, 29276005 PMC5778085

[ref21] Gao R. Penzes P. (2015). Common mechanisms of excitatory and inhibitory imbalance in schizophrenia and autism spectrum disorders. Curr. Mol. Med. 15, 146–167. doi: 10.2174/1566524015666150303003028, 25732149 PMC4721588

[ref22] Gegenhuber B. Wu M. V. Bronstein R. Tollkuhn J. (2022). Gene regulation by gonadal hormone receptors underlies brain sex differences. Nature 606, 153–159. doi: 10.1038/s41586-022-04686-1, 35508660 PMC9159952

[ref23] Giarelli E. Wiggins L. D. Rice C. E. Levy S. E. Kirby R. S. Pinto-Martin J. . (2010). Sex differences in the evaluation and diagnosis of autism spectrum disorders among children. Disabil. Health J. 3, 107–116. doi: 10.1016/j.dhjo.2009.07.001, 21122776 PMC4767258

[ref24] Greenberg M. E. Xu B. Lu B. Hempstead B. L. (2009). New insights in the biology of BDNF synthesis and release: implications in CNS function. J. Neurosci. 29, 12764–12767. doi: 10.1523/JNEUROSCI.3566-09.2009, 19828787 PMC3091387

[ref25] Hashemi E. Ariza J. Rogers H. Noctor S. C. Martínez-Cerdeño V. (2017). The number of parvalbumin-expressing interneurons is decreased in the prefrontal cortex in autism. Cereb. Cortex 27, 1931–1943. doi: 10.1093/cercor/bhw021, 26922658 PMC6074948

[ref26] Heyes S. Pratt W. S. Rees E. Dahimene S. Ferron L. Owen M. J. . (2015). Genetic disruption of voltage-gated calcium channels in psychiatric and neurological disorders. Prog. Neurobiol. 134, 36–54. doi: 10.1016/j.pneurobio.2015.09.002, 26386135 PMC4658333

[ref27] Iossifov I. Levy D. Allen J. Ye K. Ronemus M. Lee Y. H. . (2015). Low load for disruptive mutations in autism genes and their biased transmission. Proc. Natl. Acad. Sci. USA 112, E5600–E5607. doi: 10.1073/pnas.1516376112, 26401017 PMC4611648

[ref28] Jiang X. Lachance M. Rossignol E. (2016). Involvement of cortical fast-spiking parvalbumin-positive basket cells in epilepsy. Prog. Brain Res. 226, 81–126. doi: 10.1016/bs.pbr.2016.04.012, 27323940 PMC5218834

[ref29] Jurkovicova-Tarabova B. Lacinova L. (2019). Structure, function and regulation of ca(V) 2.2 N-type calcium channels. Gen. Physiol. Biophys. 38, 101–110. doi: 10.4149/gpb_2019004, 30821248

[ref30] Karimi P. Kamali E. Mousavi S. M. Karahmadi M. (2017). Environmental factors influencing the risk of autism. J. Res. Med. Sci. 22:27. doi: 10.4103/1735-1995.200272, 28413424 PMC5377970

[ref31] Lai H. C. Jan L. Y. (2006). The distribution and targeting of neuronal voltage-gated ion channels. Nat. Rev. Neurosci. 7, 548–562. doi: 10.1038/nrn1938, 16791144

[ref32] Lee E. Lee J. Kim E. (2016). Excitation/inhibition imbalance in animal models of autism Spectrum disorders. Biol. Psychiatry 81, 838–847. doi: 10.1016/j.biopsych.2016.05.011, 27450033

[ref33] Lovett-Barron M. Turi G. F. Kaifosh P. Lee P. H. Bolze F. Sun X. H. . (2012). Regulation of neuronal input transformations by tunable dendritic inhibition. Nat. Neurosci. 15, 423–430. s1-3 doi: 10.1038/nn.3024 22246433

[ref34] Ma K. McDaniel K. Webb M. Newton S. S. Lee F. S. Qin L. (2024a). A sexually dimorphic signature of activity-dependent BDNF signaling on the intrinsic excitability of pyramidal neurons in the prefrontal cortex. Front. Cell. Neurosci. 18. doi: 10.3389/fncel.2024.1496930, 39569070 PMC11576208

[ref35] Ma K. McDaniel K. Zhang D. Webb M. Qin L. (2024b). Chemogenetic inhibition of prefrontal cortex ameliorates autism-like social deficits and absence-like seizures in a gene-trap Ash1l haploinsufficiency mouse model. Genes. 15:1619. doi: 10.3390/genes15121619, 39766886 PMC11675260

[ref36] Ma K. Taylor C. Williamson M. Newton S. S. Qin L. (2023). Diminished activity-dependent BDNF signaling differentially causes autism-like behavioral deficits in male and female mice. Front. Psych. 14:1182472. doi: 10.3389/fpsyt.2023.1182472, 37205980 PMC10189061

[ref37] Manz K. M. Siemann J. K. McMahon D. G. Grueter B. A. (2021). Patch-clamp and multi-electrode array electrophysiological analysis in acute mouse brain slices. STAR Protoc. 2:100442. doi: 10.1016/j.xpro.2021.100442, 33899023 PMC8056272

[ref38] Marrocco J. Petty G. H. Ríos M. B. Gray J. D. Kogan J. F. Waters E. M. . (2017). A sexually dimorphic pre-stressed translational signature in CA3 pyramidal neurons of BDNF Val66Met mice. Nat. Commun. 8:808. doi: 10.1038/s41467-017-01014-4, 28993643 PMC5634406

[ref39] Mo K. Sadoway T. Bonato S. Ameis S. H. Anagnostou E. Lerch J. P. . (2021). Sex/gender differences in the human autistic brains: a systematic review of 20 years of neuroimaging research. Neuroimage Clin. 32:102811. doi: 10.1016/j.nicl.2021.102811, 34509922 PMC8436080

[ref40] Moechars D. Weston M. C. Leo S. Callaerts-Vegh Z. Goris I. Daneels G. . (2006). Vesicular glutamate transporter VGLUT2 expression levels control quantal size and neuropathic pain. J. Neurosci. 26, 12055–12066. doi: 10.1523/JNEUROSCI.2556-06.2006, 17108179 PMC6674853

[ref41] Murugan M. Jang H. J. Park M. Miller E. M. Cox J. Taliaferro J. P. . (2017). Combined social and spatial coding in a descending projection from the prefrontal cortex. Cell 171, 1663–77.e16. doi: 10.1016/j.cell.2017.11.002, 29224779 PMC5889923

[ref42] Pratt K. G. Aizenman C. D. (2007). Homeostatic regulation of intrinsic excitability and synaptic transmission in a developing visual circuit. J. Neurosci. 27, 8268–8277. doi: 10.1523/JNEUROSCI.1738-07.2007, 17670973 PMC6673059

[ref43] Qin L. Actor-Engel H. S. Woo M. S. Shakil F. Chen Y. W. Cho S. . (2019a). An increase of excitatory-to-inhibitory synaptic balance in the contralateral Cortico-striatal pathway underlies improved stroke recovery in BDNF Val66Met SNP mice. Neurorehabil. Neural Repair 33, 989–1002. doi: 10.1177/1545968319872997, 31524060 PMC6920539

[ref44] Qin L. Jing D. Parauda S. Carmel J. Ratan R. R. Lee F. S. . (2014). An adaptive role for BDNF Val66Met polymorphism in motor recovery in chronic stroke. J. Neurosci. 34, 2493–2502. doi: 10.1523/JNEUROSCI.4140-13.2014, 24523540 PMC3921423

[ref45] Qin L. Ma K. Wang Z. J. Hu Z. Matas E. Wei J. . (2018). Social deficits in Shank3-deficient mouse models of autism are rescued by histone deacetylase (HDAC) inhibition. Nat. Neurosci. 21, 564–575. doi: 10.1038/s41593-018-0110-8, 29531362 PMC5876144

[ref46] Qin L. Ma K. Yan Z. (2019b). Chemogenetic activation of prefrontal cortex in Shank3-deficient mice ameliorates social deficits, NMDAR hypofunction, and Sgk2 downregulation. iScience 17, 24–35. doi: 10.1016/j.isci.2019.06.014, 31247448 PMC6599088

[ref47] Qin L. Williams J. B. Tan T. Liu T. Cao Q. Ma K. . (2021). Deficiency of autism risk factor ASH1L in prefrontal cortex induces epigenetic aberrations and seizures. Nat. Commun. 12:6589. doi: 10.1038/s41467-021-26972-8, 34782621 PMC8593046

[ref48] Rogan S. C. Roth B. L. (2011). Remote control of neuronal signaling. Pharmacol. Rev. 63, 291–315. doi: 10.1124/pr.110.003020, 21415127 PMC3082452

[ref49] Rotaru D. C. Yoshino H. Lewis D. A. Ermentrout G. B. Gonzalez-Burgos G. (2011). Glutamate receptor subtypes mediating synaptic activation of prefrontal cortex neurons: relevance for schizophrenia. J. Neurosci. 31, 142–156. doi: 10.1523/JNEUROSCI.1970-10.2011, 21209199 PMC3041270

[ref50] Roth B. L. (2016). DREADDs for neuroscientists. Neuron 89, 683–694. doi: 10.1016/j.neuron.2016.01.040, 26889809 PMC4759656

[ref51] Rudy B. Fishell G. Lee S. Hjerling-Leffler J. (2011). Three groups of interneurons account for nearly 100% of neocortical GABAergic neurons. Dev. Neurobiol. 71, 45–61. doi: 10.1002/dneu.20853, 21154909 PMC3556905

[ref52] Sampath D. McWhirt J. Sathyanesan M. Newton S. S. (2020). Carbamoylated erythropoietin produces antidepressant-like effects in male and female mice. Prog. Neuro-Psychopharmacol. Biol. Psychiatry 96:109754. doi: 10.1016/j.pnpbp.2019.109754, 31454554 PMC6816335

[ref53] Sanders S. J. Campbell A. J. Cottrell J. R. Moller R. S. Wagner F. F. Auldridge A. L. . (2018). Progress in understanding and treating SCN2A-mediated disorders. Trends Neurosci. 41, 442–456. doi: 10.1016/j.tins.2018.03.011, 29691040 PMC6015533

[ref54] Sathyanesan M. Watt M. J. Haiar J. M. Scholl J. L. Davies S. R. Paulsen R. T. . (2018). Carbamoylated erythropoietin modulates cognitive outcomes of social defeat and differentially regulates gene expression in the dorsal and ventral hippocampus. Transl. Psychiatry 8:113. doi: 10.1038/s41398-018-0168-9, 29884778 PMC5993867

[ref55] Sato M. Nakai N. Fujima S. Choe K. Y. Takumi T. (2023). Social circuits and their dysfunction in autism spectrum disorder. Mol. Psychiatry 28, 3194–3206. doi: 10.1038/s41380-023-02201-0, 37612363 PMC10618103

[ref56] Satterstrom F. K. Kosmicki J. A. Wang J. Breen M. S. De Rubeis S. An J. Y. . (2020). Large-scale exome sequencing study implicates both developmental and functional changes in the neurobiology of autism. Cell 180, 568–84.e23. doi: 10.1016/j.cell.2019.12.036, 31981491 PMC7250485

[ref57] Shansky R. M. Woolley C. S. (2016). Considering sex as a biological variable will be valuable for neuroscience research. J. Neurosci. 36, 11817–11822. doi: 10.1523/JNEUROSCI.1390-16.2016, 27881768 PMC5125240

[ref58] Song M. Martinowich K. Lee F. S. (2017). BDNF at the synapse: why location matters. Mol. Psychiatry 22, 1370–1375. doi: 10.1038/mp.2017.144, 28937692 PMC5646361

[ref59] Sontheimer H. Ransom C. B. (2002). “Whole-cell patch-clamp recordings,” in Patch-Clamp Analysis: Advanced Techniques, eds. Walz W. Boulton A. A. Baker G. B. (Totowa, NJ: Humana Press), 35–67.

[ref60] Spratt P. W. E. Ben-Shalom R. Keeshen C. M. Burke K. J. Jr. Clarkson R. L. Sanders S. J. . (2019). The autism-associated gene Scn2a contributes to dendritic excitability and synaptic function in the prefrontal cortex. Neuron 103, 673–85.e5. doi: 10.1016/j.neuron.2019.05.037, 31230762 PMC7935470

[ref61] Stessman H. A. Xiong B. Coe B. P. Wang T. Hoekzema K. Fenckova M. . (2017). Targeted sequencing identifies 91 neurodevelopmental-disorder risk genes with autism and developmental-disability biases. Nat. Genet. 49, 515–526. doi: 10.1038/ng.3792, 28191889 PMC5374041

[ref62] Stoner R. Chow M. L. Boyle M. P. Sunkin S. M. Mouton P. R. Roy S. . (2014). Patches of disorganization in the neocortex of children with autism. N. Engl. J. Med. 370, 1209–1219. doi: 10.1056/NEJMoa1307491, 24670167 PMC4499461

[ref63] Street A. L. Thakkar V. P. Lemke S. W. Schoenbeck L. M. Schumacher K. M. Sathyanesan M. . (2025). Carbamoylated erythropoietin rescues autism-relevant social deficits in BALB/cJ mice. NeuroSci. 6:25. doi: 10.3390/neurosci6010025, 40137869 PMC11944669

[ref64] Tammimies K. Marshall C. R. Walker S. Kaur G. Thiruvahindrapuram B. Lionel A. C. . (2015). Molecular diagnostic yield of chromosomal microarray analysis and whole-exome sequencing in children with autism Spectrum disorder. JAMA 314, 895–903. doi: 10.1001/jama.2015.10078, 26325558

[ref65] Tremblay R. Lee S. Rudy B. (2016). GABAergic interneurons in the neocortex: from cellular properties to circuits. Neuron 91, 260–292. doi: 10.1016/j.neuron.2016.06.033, 27477017 PMC4980915

[ref66] Wang X. Bey A. L. Katz B. M. Badea A. Kim N. David L. K. . (2016). Altered mGluR5-Homer scaffolds and corticostriatal connectivity in a Shank3 complete knockout model of autism. Nat. Commun. 7:11459. doi: 10.1038/ncomms11459, 27161151 PMC4866051

[ref67] Wang T. Guo H. Xiong B. Stessman H. A. Wu H. Coe B. P. . (2016). De novo genic mutations among a Chinese autism spectrum disorder cohort. Nat. Commun. 7:13316. doi: 10.1038/ncomms13316, 27824329 PMC5105161

[ref68] Wang H. Stradtman G. G. Wang X.-J. Gao W.-J. (2008). A specialized NMDA receptor function in layer 5 recurrent microcircuitry of the adult rat prefrontal cortex. Proc. Natl. Acad. Sci. 105, 16791–16796. doi: 10.1073/pnas.0804318105, 18922773 PMC2575498

[ref69] Werling D. M. Geschwind D. H. (2013). Sex differences in autism spectrum disorders. Curr. Opin. Neurol. 26, 146–153. doi: 10.1097/WCO.0b013e32835ee548, 23406909 PMC4164392

[ref70] Yap E. L. Greenberg M. E. (2018). Activity-regulated transcription: bridging the gap between neural activity and behavior. Neuron 100, 330–348. doi: 10.1016/j.neuron.2018.10.013, 30359600 PMC6223657

[ref71] Yizhar O. Fenno L. E. Prigge M. Schneider F. Davidson T. J. O'Shea D. J. . (2011). Neocortical excitation/inhibition balance in information processing and social dysfunction. Nature 477, 171–178. doi: 10.1038/nature10360 21796121 PMC4155501

[ref72] Zhang S. Gumpper R. H. Huang X. P. Liu Y. Krumm B. E. Cao C. . (2022). Molecular basis for selective activation of DREADD-based chemogenetics. Nature 612, 354–362. doi: 10.1038/s41586-022-05489-0, 36450989

[ref73] Zhong P. Cao Q. Yan Z. (2022). Selective impairment of circuits between prefrontal cortex glutamatergic neurons and basal forebrain cholinergic neurons in a tauopathy mouse model. Cereb. Cortex 32, 5569–5579. doi: 10.1093/cercor/bhac036, 35235649 PMC9753040

[ref74] Zhong P. Qin L. Yan Z. (2020). Dopamine differentially regulates response dynamics of prefrontal cortical principal neurons and interneurons to optogenetic stimulation of inputs from ventral tegmental area. Cereb. Cortex 30, 4402–4409. doi: 10.1093/cercor/bhaa027, 32236403 PMC7325712

